# Mechanisms underlying vestibulo‐cerebellar motor learning in mice depend on movement direction

**DOI:** 10.1113/JP274346

**Published:** 2017-07-10

**Authors:** Kai Voges, Bin Wu, Laura Post, Martijn Schonewille, Chris I. De Zeeuw

**Affiliations:** ^1^ Department of Neuroscience Erasmus University Medical Center Rotterdam The Netherlands; ^2^ SINAPSE Singapore National University Singapore; ^3^ Netherlands Institute for Neuroscience Royal Netherlands Academy of Arts & Sciences Amsterdam The Netherlands

**Keywords:** climbing fibre, directionality, plasticity, purkinje cells, VOR adaptation

## Abstract

**Key points:**

- Directionality, inherent to movements, has behavioural and neuronal correlates.
- Direction of vestibular stimulation determines motor learning efficiency.
- Vestibulo‐ocular reflex gain–increase correlates with Purkinje cell simple spike potentiation.
- The locus of neural correlates for vestibulo‐ocular reflex adaptation is paradigm specific.

**Abstract:**

Compensatory eye movements elicited by head rotation, also known as vestibulo‐ocular reflex (VOR), can be adapted with the use of visual feedback. The cerebellum is essential for this type of movement adaptation, although its neuronal correlates remain to be clarified. In the present study, we show that the direction of vestibular input determines the magnitude of eye movement adaptation induced by mismatched visual input in mice, with larger changes during contraversive head rotation. Moreover, the location of the neural correlate of this changed behaviour depends on the type of paradigm. Gain–increase paradigms induce increased simple spike (SS) activity in ipsilateral cerebellar Purkinje cells (PC), which is in line with eye movements triggered by optogenetic PC activation. By contrast, gain–decrease paradigms do not induce changes in SS activity, indicating that the murine vestibulo‐cerebellar cortical circuitry is optimally designed to enhance ipsiversive eye movements.

## Key points

- Directionality, inherent to movements, has behavioural and neuronal correlates.
- Direction of vestibular stimulation determines motor learning efficiency.
- Vestibulo‐ocular reflex gain–increase correlates with Purkinje cell simple spike potentiation.
- The locus of neural correlates for vestibulo‐ocular reflex adaptation is paradigm specific.

**Abbreviations**
CEM: compensatory eye movement
CS: complex spikes
PFA: paraformaldehyde
LTD: long‐term depression
LTP: long‐term potentiation
n–t: naso‐temporal
OKR: optokinetic reflex
PC: Purkinje cell
PF: parallel fibres
SS: simple spikes
t–n: temporo‐nasal
VOR: vestibulo‐ocular reflex
VVOR: visual vestibulo‐ocular reflex

## Introduction

As a result of the structure and connections of their receptors, afferents from the vestibular and visual system carry inherently direction‐selective information (Ezure & Sasaki, 1978; Lopez‐Barneo *et al*. 1982). In principle, this type of information may be relevant for cerebellar motor learning, such as goal‐directed adaptation of reaching tasks of the limbs, smooth pursuit eye movements or compensatory eye movements (CEMs) (Robinson, 1976; Distler & Hoffmann, 2003; Smith & Shadmehr, 2004; Tseng *et al*. 2007; Cahill & Nathans, 2008; Medina & Lisberger, 2008). Sensory and motor information required for motor learning is conveyed to the cerebellar cortex through two main afferent systems (Gao *et al*. 2012). These include the mossy fibre system, which modulates simple spike (SS) activity of Purkinje cells (PCs) via parallel fibres (PFs) and interneurons, and the climbing fibre system, which gives rise to the all‐or‐none complex spike (CS) activity of PCs (Gao *et al*. 2012). The PF to PC synapse, as well as the PF to molecular layer interneuron synapse and molecular layer interneuron to PC synapse, form major sites of cerebellar integration and plasticity (Lev‐Ram *et al*. 2002; Coesmans *et al*. 2004), all of which are putatively controlled by the climbing fibre input (Gao *et al*. 2012). Early, postsynaptic long‐term depression (LTD) has been suggested as a possible mechanism (Ito, 1982; Ito & Kano, 1982) to adjust the efficacy of these synapses, although to what extent postsynaptic long‐term potentiation (LTP) also contributes to a given behaviour is still under debate (Hansel *et al*. 2006; Medina & Lisberger, 2008; Schonewille *et al*. 2010, 2011; Peter *et al*. 2016; Gutierrez‐Castellanos *et al*. 2017). Because spiking activity of the climbing fibres is highly direction‐selective (Simpson & Alley, 1974), the emerging hypothesis is that plasticity mechanisms contributing to learning are direction‐selective too. Hence, cerebellar learning, in terms of mechanism and strength, may in principle also depend on the direction of sensory input and/or desired motor output. However, for adaptation of the vestibulo‐ocular reflex (VOR), which is one of the most studied cerebellar motor learning tasks (Lisberger & Fuchs, 1978; Hirata & Highstein, 2000; Ito, 2006; Ushio *et al*. 2011), the efficacy of direction‐specific learning has not been assessed in detail yet and the quantitative relation between movement direction and adaptation still remains to be studied in the same experiments (Ushio *et al*. 2011; Migliaccio & Schubert, 2013; Nguyen‐Vu *et al*. 2013). More specifically, the contribution of both SS and CS activity of PCs to adaptation of the VOR remains enigmatic (Wulff *et al*. 2009; Nguyen‐Vu *et al*. 2013; Kimpo *et al*. 2014; Badura *et al*. 2016; Titley and Hansel, 2016). Based on previous studies in mutants in which various forms of plasticity and/or parts of the cerebellar cortical network are affected (Schonewille *et al*. 2010; Boyden *et al*. 2006, 2011; Gao *et al*. 2012; Galliano *et al*. 2013), several predictions can be made on potential direction‐dependent plasticity mechanisms, even with data that have been collected following sinusoidal stimulations. Deficits in potentiation mechanisms in PCs robustly impair VOR gain–increase, although with a lesser effect on gain–decrease paradigms (Schonewille *et al*. 2010), whereas selective deficits in LTD at the parallel fibre to PC synapse do not appear to lead to obvious changes in any form of VOR adaptation (Schonewille *et al*. 2011). Interestingly, in the related Purkinje cells (vertical‐axis type; see Methods) increasing SS activity and thus decreasing CS activity is known to correlate with naso‐temporal (n–t) eye movements, during both the VOR and optokinetic reflex (OKR) evoked by visual input (Graf *et al*. 1988; De Zeeuw *et al*. 1995; Schonewille *et al*. 2006). Taken together, this would imply that the strongest training paradigms include those that occur when SS activity of PCs is enhanced and when the climbing fibres are inhibited (i.e. during contraversive head movements) (De Zeeuw *et al*. 1995; Badura *et al*. 2013, 2016). Hence, we hypothesize that gain–increase and gain–decrease trainings can both be optimally executed during contraversive head movements and that the levels of increase and decrease of both SS and eye movement gain depend on the level of concomitant climbing fibre activity, with low climbing fibre activity leading to gain–increases and vice versa because of the windows of opportunity set by the synergistic forms of plasticity (Gao *et al*. 2012).

In the present study, we use direction‐specific sigmoidal, rather than sinusoidal, visual and vestibular, to assess the contribution of individual movement directions in vestibulo‐cerebellar learning. By simultaneously monitoring eye movement performance and corresponding PC activity during VOR adaptation, we test our hypothesis that cerebellar learning is direction‐dependent. We show that adaptation to the visual stimulus is more pronounced during contraversive head movements, both for gain–increase and gain–decrease VOR adaptation. The learned changes during contraversive head movements are reflected in the activity of PCs when we increase the amplitude of the VOR gain but not when we decrease it. Finally, optogenetic stimulation of PCs confirms the presence of a quantitative, causal relationship between SS firing, movement direction and amplitude.

## Methods

### Ethical approval

All experiments were performed under the GGO license no. IG 04‐197 and were approved by the Dutch animal ethical committee (DEC, EMC 2572).

#### Animals

Wild‐type mice (*n* = 80; C57BL/6, Envigo, Venray, The Netherlands; food/water available *ad libitum*) were prepared for eye movement recordings under general inhalation anaesthesia (isoflurane, in 100% O_2_; 4% induction, 1.5–2.0% maintenance; Pharmachemie BV, Haarlem, The Netherlands) with a magnetic pedestal that was attached to the frontal and parietal bones of the skull with dental cement (Charisma; Heraeus Kulzer, Hanau, Germany). Mice received buprenorphine (Temgesic, 50 μg kg^−1^, i.p.; Reckitt Benckiser Pharmaceuticals Ltd, Slough, UK) and carprofen (Rimadyl, 1 mg kg^−1^, i.p., Zoetis, Parsippany‐Troy Hills, NJ, USA) pre‐ and/or postsurgery for analgesia and prevention of inflammation, respectively. Wild‐type mice (*n* = 30) from the same supplier were prepared under the same conditions with a magnetic pedestal and a craniotomy in the occipital bone to give access to the cerebellar cortex for extracellular recordings. To protect the brain, the dura was left intact, and a chamber made out of dental cement (Simplex Rapid; Kemdent, Swindon, UK) was built around the craniotomy, and closed with ointment (Duratears Z; Alcon Inc., Fort Worth, TX, USA) and bone wax (Ethicon W810; Johnson & Johnson International, New Brunswick, NJ, USA). After surgery, mice were allowed to recover for at least 5 days. After the experiments, animals were first anaesthetized as described above and then killed by cervical dislocation. For optogenetic experiments, mice expressing Cre recombinase under the Purkinje cell specific L7 promotor (L7Cre) (Barski *et al*. 2000) were crossed with mice carrying floxed hChR2(H134R)‐tdT (Ai27D) to obtain L7‐Ai27D animals that express channelrhodopsin‐2 H134R fused with TdTomato (Madisen *et al*. 2012) in cerebellar Purkinje cells (*n* = 4; Jackson Laboratories, Bar Harbor, ME, USA).

#### Stimulation

Vestibular stimulation was delivered through a turntable (diameter 60 cm) with the head of the mouse fixed in the centre and its body placed in a custom‐made mouse restrainer. For visual stimulation, a high‐resolution (3800 × 1000 pixels) rear projection system was used. Three standard projectors (D3Series; Vivitek, Hoofddorp, The Netherlands) projected a blended and warped (Vioso Presenter; Vioso GmbH, Duesseldorf, Germany) image, consisting of a random dotted pattern (minimum shape size: 2°; colours: black and white) onto a dome‐shaped screen (radius: 35 cm; azimuth: 240°; elevation: −10 to +60°). Two sinusoidal functions were combined to generate a sigmoidal output for the vestibular stimulus (custom sequencer file; Spike2; Cambridge Electronic Design, Cambridge, UK). The rotation of the table was read out via a potentiometer fixed at the turning axis of the table. Pre‐rendered content for the projection system was calibrated with a laser pointer and a webcam mounted on the turntable (custom functions; Matlab 2012a; MathWorks Inc., Natick, MA, USA) (see Supporting information, Movies S1 and S2).

Mice were familiarized with the experimental set‐up 3 days prior to the experiments by providing visual and vestibular stimulations for 30 min. Experiments started with recordings of baseline CEMs: OKR, visual vestibulo‐ocular reflex (VVOR) and VOR (stimulation amplitudes: 5, 10, 15, 20, 25 and 30°, with peak velocities 3.8, 7.9, 11.3, 14.6, 18.3 and 22.0 ° s^−1^), either in clockwise or counter clockwise direction. VOR gain adaptation experiments started on the next day, beginning with an initial VOR recording. VOR gain–increase and gain–decrease were induced by training mice in blocks (50 repeats, each stimulus cycle is 12 s = 10 min) consisting of out‐of‐phase or in‐phase vestibular and visual input (both at 10° amplitude), respectively, and directly followed by probe trials (20 repeats, 4 min), consisting of VOR recordings in the dark (also at 10° amplitude). This block was repeated five or six times (i.e. massed training without intervals, total duration 74 or 88 min). Some mice were trained with more than one paradigm, interleaved by a recovery period of at least 7 days, during which mice were kept under a 12:12 h light/dark cycle.

VOR gain adaptation experiments combined with electrophysiological recordings started with an initial VOR recording, followed by a block of training sessions (2 × 10 repeats) and probe trials (1 × 10 repeats). This block was repeated three times (total duration 20 min). In between each experiment, mice recovered for at least 24 h under a 12:12 h light/dark cycle. Out of the 32 cells, four were stable only until the third probe trial repeat, which was then taken as the last probe trial for further analysis.

#### Eye movement recordings

Eye movements were recorded and calibrated with a video camera‐based system (ETL‐200; iScan; Illumina, San Diego, CA, USA) as described previously (Stahl *et al*. 2000). Three infrared emitters (maximum output 600 mW, dispersion angle 7°, peak wavelength 880 nm) were used to illuminate the eye: two from below that were fixed to the table and one from above, which was fixed to the camera and of which the corneal reflection was used as the reference point. To allow mice an unobstructed view onto the screen, a hot mirror was mounted between the mouse eye and camera. To achieve natural recording conditions during VOR measurements, we used the contrast‐free, Ganzfeld character of the projection screen under ambient light conditions (see Supporting information, Movie S1). Finally, we also performed experiments in which pilocarpine (pilocarpine nitrate 2%; Bausch & Lomb, Rochester, NY, USA) was applied to the recorded eye and experiments in which the recordings were performed in complete darkness, allowing assessment of the impact of the different contexts; these experiments involved successive training paradigms.

#### Electrophysiology

Glass pipettes (outer diameter 1.5 mm, outer diameter 0.86 mm, borosilicate; Sutter Instruments, Novato, CA, USA) were pulled (P‐1000; Sutter Instruments) and filled with 2 m NaCl (tip diameter: 2 μm; impedance: 2–4 MΩ). Extracellular recordings were amplified, filtered and sampled (gain: 100; high pass: 30 Hz; low pass: 10 kHz; rate: 50 kHz) (Axon Multiclamp 700B and Digidata 1440A; Molecular Devices, Sunnyvale, CA, USA). Signals from eye movement recordings, stimuli and PCs were combined (power1401; Cambridge Electronic Design) and stored on hard disc for offline analysis. Pipettes were mounted on a digital 3‐axis drive (SM‐5; Luigs Neumann, Ratingen, Germany) and lowered onto the left paramedian lobule after the dura was removed under light local anaesthesia (Xylocaine; AstraZeneca, Cambridge, UK). An angled (−30° azimuth from the rostro‐caudal midline; elevation −46°) approach made it possible to reach the flocculus at a depth of 3.5–4.5 mm.

PCs (*n* = 67 from C57BL/6 mice and *n* = 20 from L7‐Ai27 mice) were identified by the presence of simple and complex spikes, and determined to be from a single unit by confirming that each complex spike was followed by a climbing fibre pause. To ensure recordings were made from PCs in floccular vertical‐axis zones (visual stimulus rotation axis azimuth: 0°; elevation: 90°) (Schonewille *et al*. 2006), the phase locking of CSs was compared between sinusoidal stimulation around the vertical axis and horizontal axis (horizontal axis here refers to the axis at 45° azimuth from the rostro‐caudal midline; with an elevation of 0°). If CSs showed strong phase locking when the visual stimulus moved temporo‐nasally, the zone was identified as a vertical‐axis zone. If phase locking was strongest during upward movement of the stimulus, the zone was identified as a horizontal‐axis zone. For the cells that were stable after experiments (*n* = 16), a tuning curve with sinusoidal rotations in the vertical axis, horizontal axis and an intermediate angle (azimuth: 45°; elevation: 45°) was recorded and the coefficient of synchronization and the z value were calculated.

#### Optogenetics

In L7‐Ai27D (see above), an additional small craniotomy in the periotic capsule with a diameter of ∼800 μm was performed. To form a cannula that gives access to the caudal floccular complex, the tip of a plastic pipette (5 mm) was placed perpendicular to the bone and fixed with dental cement (Simplex Rapid; Kemdent). After a recovery period of at least 5 days, an optical fibre (200 μm × 5 mm, filter: 470 nm, driver: LEDD1B; Thorlabs, Ely, UK) was lowered between 4 and 5.5 mm into the cannula, and placed on the outside of the paraflocculus, directed towards the flocculus. To mimic eye movements and spiking patterns during visual stimulation, the LED was pulsed over a period of 2.25 s, with increasing frequencies (60, 80, 90, 100 and 120 Hz) and a duty cycle of 50%. Irradiance was varied between 0.12, 0.42, 0.72 and 1.8 mW mm^−2^ measured at the electrode tip prior to experiments (Optical power meter 1830‐C, with sensor 883‐SL; Newport, Irvine, CA, USA).

#### Analysis

Eye movement position traces were analysed with a custom plugin for SpikeTrain, a multipurpose *in vivo* analysis tool (Neurasmus BV, Rotterdam, The Netherlands). Fast eye movement components were detected in the calculated velocity traces and automatically removed from the position and velocity signal. The movement amplitude was calculated by fitting a sigmoidal function to the average eye position and extracting coefficient a from (1).

(1) $$f\left(t\right)=\frac{a}{e^{-b^{*}\left(t-c\right)}}$$

Gain was calculated as the ratio between stimulus amplitude and eye movement amplitude. Eye movement delay was calculated as the time shift between the velocity peak of the stimulus and the velocity peak of the eye movement. Extracellular recordings were spike sorted with SpikeTrain (Neurasmus BV). Using superparamagnetic clustering, SSs and CSs usually split up in two clusters (i.e. <0.1) (Quiroga *et al*. 2004). Spikes during fast eye movement components were removed in a window of 40 ms prior to 80 ms post movement onset. If a stimulus repeat contained movement artefacts or individual eye movements diverging more than 30% from the average, this repeat was manually excluded from further analysis. Basic spiking characteristics such as firing rate, CV and CV2 were calculated over the whole recording to ensure stable recording conditions (data not shown). Average spiking rates for individual parts of the stimulus were calculated from peristimulus time histograms (bin size for complete stimulus of 12 s: SSs 100 ms, CSs 300 ms; bin size for segments of 4.5 s: SSs 50 ms, CSs 100 ms). Averages of histograms for eye movements and spiking data in figures were smoothed with a running average (span: eye movements 5, SSs 10, CSs 3). Heat maps were derived from the convoluted raster plots (Gaussian filter width/sigma: SSs 400/80, CSs 200/20).

To determine position, velocity and acceleration sensitivity of PC spike modulation, eye movements were fitted to the PSTHs of SSs and CSs using:

(2) $$f\left(t\right)=k*f\left(t\right)+r*f\left(\dot{t}\right)+u*f\left(\ddot{t}\right)+c$$

where *k* gives the position, *r* is the velocity and *u* is the acceleration dependence. For data following sigmoidal stimulation, eye movements were fitted with formula (1) and eye movement data following optogenetic stimulation were linearly fitted. Latencies of peak and trough spike modulation were retrieved from the fitted functions at the maximum or minimum point, respectively.

#### Histochemistry

After recordings, L7Cre/Ai27D mice were deeply anaesthetized with nembutal and perfused with 75 ml of 4% paraformaldehyde (PFA). The brains were removed from the skull and postfixed for 1–2 h in 4% PFA, and stored in 0.1 m phosphate buffer containing 10% sucrose. After embedding in 10% gelatin and 10% sucrose, blocks were hardened in a solution containing 10% formaldehyde, 30% sucrose for 1–2 h at room temperature and then stored overnight in 0.1 m phosphate buffer with 30% sucrose at 4°C. Coronal sections with a thickness of 40 μm were made with a sliding microtome with cryostate adaptations. For fluorescence, the sections were stained with 4',6‐diamidino‐2‐phenylindole and mounted on cover slips, dried and covered with vectashield (Vector‐H‐1000; Vector Laboratories, Inc., Burlingame, CA, USA). The expression of Ai27 was determined using a Zeiss LSM700 confocal laser scanning microscope with 10×, 20× and 63× (oil immersion) objectives.

#### Statistical analysis

Eye movement gains in the two directions were compared using a linear mixed model with the trial number as repeated measure, a diagonal covariance matrix and fixed effects for the trial number, the movement direction and uni‐ or bidirectional stimulation. Although this analysis method also allows for the comparison of mean values in between trials of one group resulting in a *P* value for each repeat, we report only the *F* and *P* values over all repeats. Spiking data had an additional repeated measure when spontaneous and stimulus evoked spiking frequencies or the initial and the secondary segment were compared. To compare the depth of modulation of spiking data, the spontaneous firing frequency was subtracted from the stimulus evoked firing frequency and a linear mixed model was set‐up, comparable to that for eye movement gains. Significance of correlations was tested with Spearman's rank correlation coefficient. All statistical analysis was conducted with SPSS, version 21 (IBM Corp., Armonk, NY, USA).

## Results

To evoke and adapt direction‐specific compensatory eye movements, we subjected mice to visual and vestibular stimuli around an earth‐vertical axis with a sigmoidal position profile. By contrast to sinusoidal stimulation, a sigmoidal stimulus temporally separates the contribution of the individual movement directions and allows a more direct correlation of spiking activity to eye movements. We recorded eye movements from the left eye and simultaneously recorded single cell activity of PCs (total *n* = 87) from the left flocculus of mice (eye movements only, *n* = 68; electrophysiology and eye movements, *n* = 30).

### Preferred directions for baseline visual compensatory eye movements

**Figure 1:**
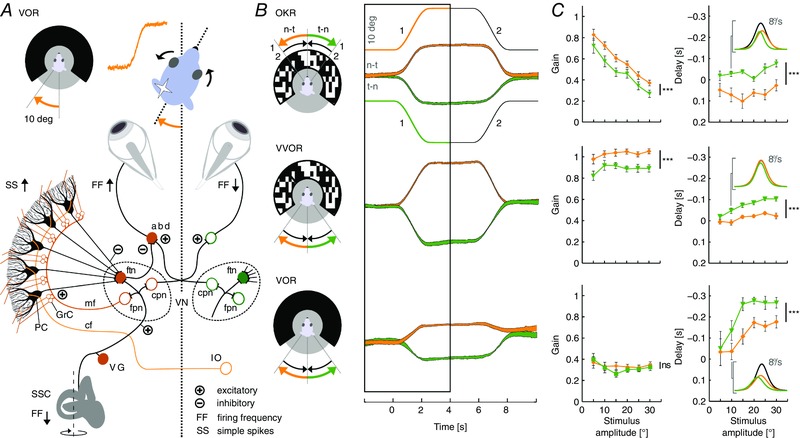
Baseline compensatory eye movements have a preferred direction *A*, rotating a head‐fixed mouse in the dark in a contraversive direction with respect to the recording side will evoke a compensatory eye movement in the n–t direction towards the ipsilateral visual field (top) via a three‐neuron arc in the brainstem (bottom, filled orange symbols). Sensory information from the semi‐circular canals (SCC), transferred by the vestibular ganglion cells (VG), reaches the vestibular nuclei (VN). Excitatory inputs drive flocculus projection neurons (fpn), flocculus target neurons (ftn) and commissural projection neurons (cpn). The PCs in the flocculus receive their input from the VN via mossy fibres (mf) to the granule cells (GrC) and, after processing in the molecular layer, provide an inhibitory input to the VN, which in turn inhibits ipsilateral abducens neurons (abd); thus, through a process of disinhibition, PCs are expected to eventually facilitate an n–t eye movement. The climbing fibres (cf) of the inferior olive (IO) provide the error signal to the contralateral PCs. *B*, CEMs following direction‐selective stimulation. Icons (left) display eye movement (n–t orange, t–n green) and stimulus direction (arrow heads) for the visual (arrows top) and the vestibular (arrows bottom) stimulus. Numbers at the icon for OKR indicate the first and second stimulus segment. Note that visual stimulation evokes CEMs in the same direction as the stimulus, whereas vestibular stimulation‐evoked movements are opposite to the direction of the stimulus. Right, stimulus (thin lines) and average eye movement traces recorded at a stimulation amplitude of 10° for n–t and t–n eye movements. Analysis of eye movement was performed during the initial segment (black box, number 1). *C*, gains of average eye movements (left) were significantly larger in the n–t direction (*n* = 20) than in the t–n (*n* = 19) direction for the visually driven OKR and VVOR but not for the VOR over stimulation amplitudes ranging from 5 to 30° (peak velocities 3.8 to 22.0 ° s^−1^). Delays (right) for the t–n direction were leading (i.e. have a more negative delay than) those of the other direction. Insets show example velocity profiles of n–t and t–n movements following 10° stimulation (black lines). Data are the mean ± SEM; population data (*B*, middle) represent the mean ± SEM.

We first investigated to what extent normal, un‐adapted eye movement reflexes reveal a preference for movement direction (Fig. 1 *A*). Eye movements evoked by sigmoidal horizontal visual stimulation at amplitudes ranging from 5 to 30° (i.e. peak velocities 3.8 to 22.0 ° s^−1^; *n* = 32 mice) showed a clear direction preference (Fig. 1 *B*; see also Supporting information, Movie S1). Independent of stimulus amplitude, the preferred direction for visually‐driven eye movements was the n–t direction (i.e. for the optokinetic reflex, OKR, and the visual vestibulo‐ocular reflex, VVOR, but not VOR) (Fig. 1 *C*). For the solely visually‐driven OKR, gains decreased with increasing stimulus amplitude, yet eye movements in the n–t direction consistently yielded higher gains than those in the opposite direction (*n* = 39, mixed model: *P* < 0.001, *t* = 4.81) (Fig. 1 *C*). Interestingly, the OKR peak velocity was delayed in the n–t direction with respect to that in the temporo‐nasal (t–n) direction (*P* < 0.001, *t* = 7.05) (Fig. 1 *C*). Similarly, VVOR gain values showed a preference for n–t movements (*n* = 39, *P* < 0.001, *t* = 8.66) and delays of VVOR peak velocity in the n–t direction were also significantly longer than those in the t–n direction (*P* < 0.001, *t* = 8.72) (Fig. 1 *C*). For the solely vestibularly driven VOR (Fig. 1 *B* and *C*), gains were not significantly different for the two directions (*n* = 31, *P* = 0.10, *t* = 1.64) but, in line with the VVOR data described above, VOR delays were shorter for the eye movements in the t–n direction (*P* < 0.001, *t* = 6.20).

**Figure 2:**
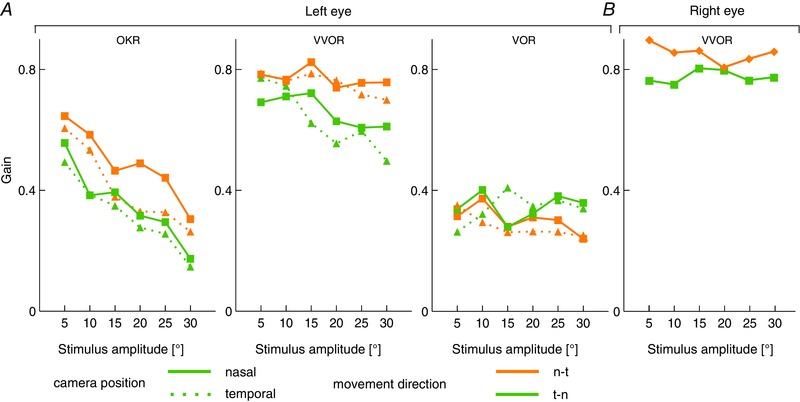
Relation eye movement gain, preferred direction and camera position *A*, to rule out the possibility that camera position relative to the eye might have resulted in asymmetric recording conditions, eye movements were measured at two additional camera positions: at +10° and −10° from the usual position. Independent of camera position, eye movements during visual stimulation were larger in the n–t direction. *B*, moreover, again, gains did not show any direction dependence during vestibular stimulation in the dark. To further ensure that differences in preferred direction are not related to the camera position or stimulation, the same experiments were repeated for the right eye (right) showing the same preferrence for the n–t direction. Data are the mean values.

To ensure that the preferred direction was independent of the camera position, we tested baseline CEMs with extreme camera positions, ±10° towards nasal and temporal from the centre position. Consistent with the data described above, gains were higher (*n* = 8, mixed model: OKR, temporal cam position, n *vs*. t: *P* < 0.001, *t* = 3.56; nasal cam position, n *vs*. t: *P* < 0.001, *t* = 4.74) in the n–t direction for visually driven CEMs but not for VOR (Fig. 2 *A*). These eye movement recordings were all obtained from the left eye. To confirm that directional preference is indeed n–t and not a right–left preference, we subsequently also evaluated the direction selectivity in the right eye (*n* = 13) (Fig. 2 *B*). Here, direction‐selectivity largely reversed from right to left, to left to right, indicating that the preference is predominantly related to the preference for n–t eye movements (mixed model: VVOR, n *vs*. t: *P* < 0.001, *t* = −2.11).

Thus, in comparison to sinusoidal stimulation (De Zeeuw *et al*. 1995; Wulff *et al*. 2009), the visually driven CEMs following sigmoidal stimulation showed similar eye movement‐stimulation amplitude relations, with velocity profiles of OKR lagging behind, profiles of VVOR closely tuned to the velocity profile of the stimulus, and VOR velocity profiles leading that of the stimulation. Moreover, and most importantly, both types of visually driven CEMs (i.e. OKR and VVOR) showed higher gain values towards the ipsilateral visual field.

### Preferred directions for PC activity

**Figure 3:**
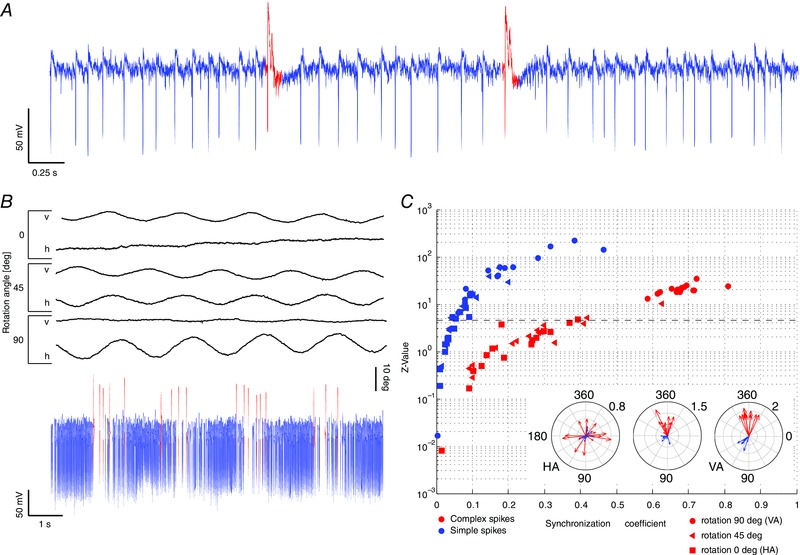
Identification of vertical‐axis floccular PCs *A*, *in vivo* extracellular recordings were obtained from cerebellar PCs during visual and/or vestibular stimulation in awake mice. Recordings were identified as a single unit by the consistent presence of a pause in SSs (blue) following each CS (red). *B*, traces show examples of vertical axis (VA) and horizontal axis (HA) eye movements following visual stimulation around three different rotational axes (from top to bottom: HA, intermediate and VA). To separate VA‐responsive PCs from HA‐responsive and non‐floccular PCs, the activity of PCs in response to each stimulus was recorded and SSs (blue) and CSs (red) were identified (bottom). *C*, PCs showing significant (*P* < 0.001 for z values > 4.6) synchronization with VA but not HA stimulus were identified by the use of circular statistics and used for further analysis. Insets: polar plots show a strong phase relation and depth of modulation for SSs and CSs during VA stimulation (right) but less so during intermediate or vertical axis stimulation (middle and left, respectively).

**Figure 4:**
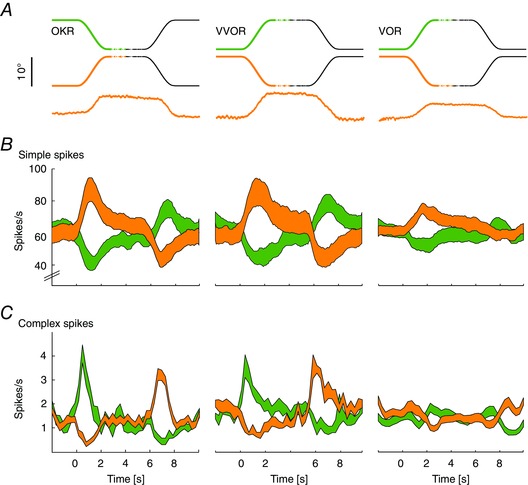
PCs spiking patterns reflect the direction of movement *A*, stimulus traces in the two movement directions (top, n–t orange, t–n green) and example eye movement traces (bottom, n–t direction). Note that data are sorted according to eye movement, rather than stimulus, direction. *B* and *C*, average SS and CS firing during baseline CEMs measured at a stimulation amplitude of 10°. PCs (total of 27) showed an increase or decrease of SS firing and the reciprocal CS pattern for the n–t and t–n direction, respectively. *B*, SS firing pattern in one direction was a mirror image of the spiking pattern in the other direction in terms of depth of modulation and latency to peak modulation. *C*, visually driven CSs modulated stronger in the t–n direction with a bigger lead of peak modulation compared to the n–t direction; the CSs during stimulation in t–n direction may explain why eye movement delays were shorter in this direction. CS activity during VOR showed a bimodal pattern with an initial increase followed by a decrease in the n–t and vice versa for the t–n direction. Data are the mean ± SEM; population data (*B* and *C*) represent the mean ± SEM.

Next, we characterized the correlation of PC activity with different movement directions. Accordingly, floccular vertical‐axis PCs (*n* = 27 cells; 11 mice) were identified by the presence of CSs and SSs (Fig. 3 *A*), the consistent presence of a pause in SS firing after a CS (i.e. climbing fibre pause) and optimal CS responses during a contraversive visual stimulus around the vertical axis (Fig. 3 *B* and *C*) (Simpson & Alley, 1974; De Zeeuw *et al*. 1995; Schonewille *et al*. 2006; Winkelman *et al*. 2014). Vertical‐axis PCs had an average baseline SS firing frequency of 63 ± 2 spikes s^−1^ at rest (Table 1) (Fig. 4 *A* and *B*).

**Table 1:** SS firing rates during baseline compensatory eye movements in the n–t and t–n direction

		n–t			t–n		
		Baseline	Peak/trough	Depth	Baseline	Peak/trough	Depth
OKR	Segment 1	59 ± 5	88 ± 8	27 ± 3	59 ± 3	35 ± 3	−23 ± 2
	Segment 2		49 ± 6	−13 ± 3		69 ± 5	9 ± 4
VVOR	Segment 1	59 ± 3	80 ± 7	20 ± 3	62 ± 4	40 ± 4	−22 ± 2
	Segment 2		53 ± 4	−9 ± 4		69 ± 6	10 ± 4
VOR	Segment 1	66 ± 6	76 ± 7	9 ± 1	74 ± 6	64 ± 8	−9 ± 1
	Segment 2		63 ± 5	−3 ± 2		72 ± 5	2 ± 2

Baseline values were calculated from the periods preceding the start of stimulation. Peak and trough rates refer to the maximum and minimum modulation during stimulation, respectively, whereas depth equals the difference between peak modulation and baseline firing. Segments 1 and 2 refer to the first and the second movement direction segment of the stimulus, respectively (spikes s^−1^; mean ± SEM; for further explanation, see Fig. 1 *B*).

The depth of modulation (i.e. the change in spike rate relative to baseline) of SSs was significantly greater during OKR and VVOR than that during VOR (SS depth of modulation OKR *vs*. VOR: *P* < 0.001, *t* = −5.346; VVOR *vs*. VOR: *P* = 0.003, *t* = −3.586), but for all three types of CEMs, SS firing rate increased during n–t and decreased during t–n movements of the ipsilateral eye (Fig. 4 *B* and *C*). Latencies of the maximum change in SS modulation with respect to the maximum change in stimulus velocity were not different for the two movement directions for any type of CEM (SS latency OKR n–t: −0.01 ± 0.09 s, t–n: −0.14 ± 0.09 s; VVOR n–t: 0.02 ± 0.07 s, t–n: −0.13 ± 0.11 s; VOR t–n: −0.07 ± 0.11 s, n–t: −0.16 ± 0.13 s; all *P* > 0.2) and maximum SS modulation generally preceded maximum velocity peak of eye movements (Fig. 1 *C*). Interestingly, for all paradigms, SS modulation during the first segment of the stimulus cycle (coloured parts of stimulus traces) was significantly greater than that of the second segment (OKR: *P* < 0.001, *t* = 9.52; VVOR: *P* = 0.001, *t* = 4.34; VOR: *P* = 0.042, *t* = −2.18). Because the first half of the stimulation placed the ipsilateral eye at an eccentric position at the start of the second half of the visual and/or vestibular stimulation, these data suggest that absolute eye position influences PC SS activity, and hence all subsequent reported data were obtained during the first half of stimulation (Figs 1 *B* and 4 *B*).

The reciprocal SS and CS activity of vertical‐axis PCs during visual and/or vestibular sigmoidal stimulation showed significantly higher levels of modulation during OKR and VVOR than VOR (OKR *vs*. VOR: *P* < 0.001, *t* = 4.183) (Table 2) (Fig. 4 *C*). Interestingly, the depth of CS modulation during OKR was not significantly different from that during VVOR (n–t: *P* = 0.66, *t* = 0.45; t–n: *P* = 0.64, *t* = 0.48), despite the fact that OKR gains and delays were significantly lower and longer, respectively, than those during VVOR (gain: n–t, *P* < 0.001, *t* = −23.766 and t–n, *P* < 0.001, *t* = 4.182; delay: n–t, *P* < 0.001, *t* = 7.663 and t–n, *P* < 0.001, *t* = −21.841) (Fig. 1 *C*). Latencies of CS peak modulation in the t–n direction were consistently shorter than in the n–t direction (OKR: *P* = 0.03, *t* = 2.18; VVOR: *P* = 0.03, *t* = −2.21), in line with the shorter delay in peak velocity of the eye movement (Fig. 1 *C*). During VOR, the CS modulation pattern diverged even more from that of the SSs and revealed a bimodal spiking pattern, consisting of an initial increase followed by a decrease in firing when the ipsilateral eye moved into n–t direction and a reversed pattern in the t–n direction (in 10 out of 16 cells). Together, these data indicate that CS modulation during visuo‐vestibular sigmoidal stimulation encodes mainly retinal slip and in addition probably some vestibular and/or motor signals (De Zeeuw *et al*. 1995; Winkelman *et al*. 2014).

**Table 2:** CS firing rates during baseline compensatory eye movements in the n–t and t–n direction

		n–t			t–n		
		Baseline	Peak/trough	Depth	Baseline	Peak/trough	Depth
OKR	Segment 1	1.4 ± 0.1	1 ± 0.1	−0.4 ± 0.1	1.5 ± 0.2	3.1 ± 0.3	1.5 ± 0.2
	Segment 2		2.8 ± 0.2	1.4 ± 0.1		0.7 ± 0.2	−0.7 ± 0.1
VVOR	Segment 1	1.4 ± 0.1	0.9 ± 0.2	−0.5 ± 0.1	1.4 ± 0.1	2.8 ± 0.3	1.3 ± 0.1
	Segment 2		1.8 ± 0.2	0.6 ± 0.2		0.8 ± 0.2	−0.8 ± 0.1
VOR	Segemnt 1	1.4 ± 0.1	1.5 ± 0.1	0.2 ± 0.1	1.2 ± 0.2	1.01 ± 0.20	−0.2 ± 0.1
	Segment 2		1.15 ± 0.03	−0.2 ± 0.1		1.0 ± 0.3	−0.3 ± 0.2

Values for baseline, peak and depth were calculated as described in Table 1 (spikes s^−1^; mean ± SEM).

### Magnitude of VOR gain adaptation depends on movement direction

**Figure 5:**
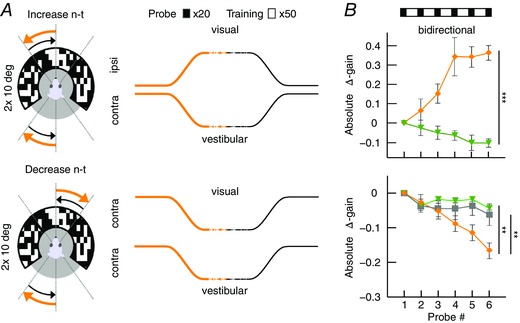
VOR gain–increase and gain–decrease training has a principal learning direction *A*, scheme of stimulation during gain–increase (vestibular and visual stimulus out‐of‐phase, top) and gain–decrease training (vestibular and visual stimulus in‐phase, bottom). Icons depict stimulation (left) and stimulus traces (right) during training sessions with contraversive vestibular stimulation (n–t, orange, cf. Fig. 6 *A* for unidirectional ipsiversive stimulation); during probe trials, mice received vestibular stimulation in the dark in the respective direction. *B*, changes in eye movement gain were measured during VOR probe trials (black boxes; 20 repeats = 4 min, top), caused by five gain–increase (*n* = 24; starting gain: 0.35 ± 0.02) (top) or gain–decrease (*n* = 26; starting gain: 0.35 ± 0.03) (bottom) training sessions (white boxes; 50 repeats = 10 min; total: 6 × 4 mins + 5 × 10 mins = 74 mins). Learning effect depended on the direction of stimulation, with significantly better learning in trainings based on contraversive (n–t, orange) than with ipsiversive (t–n, green) vestibular stimulation. Note that this difference was independent of whether the visual stimulus was presented bidirectionally (i.e. present during both ipsi‐ and contraversive vestibular input) or unidirectional, when the visual stimulus was presented only during the first segment of the vestibular stimulus (i.e. only during either ipsi‐ or contraversive input) (Fig. 6). VOR habituation in the learning direction (grey line, bottom left) (i.e. contraversive head rotations) is significantly different from VOR decrease in the same direction. Data are the mean ± SEM.

**Figure 6:**
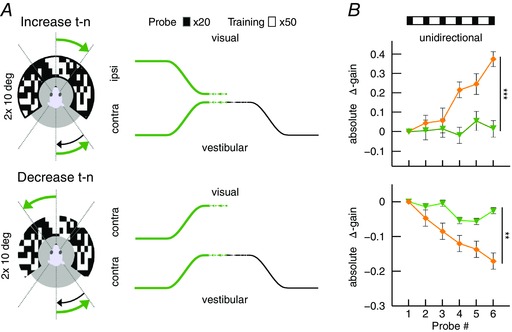
Unidirectional VOR gain adaptation *A*, schemes showing how unidirectional stimulation was provided during gain–increase (vestibular and visual stimulus out‐of‐phase, top) and gain–decrease training (vestibular and visual stimulus in‐phase, bottom). Icons depict visual and vestibular stimulation (left) and stimulus traces (right) during training sessions with ipsiversive vestibular stimulation (t–n, green); during probe trials, mice received vestibular stimulation in the dark in the direction indicated in black. *B*, changes in eye movement gain were measured during VOR probe trials (black boxes; 20 repeats = 4 min, top); these changes were induced by five gain–increase (top) or gain–decrease (bottom) training sessions (white boxes; 50 repeats = 10 min; total time 74 min (6 × 4 mins + 5 × 10 mins). As during bidirectional stimulation, the learning effect depended on the direction of stimulation, with significantly better learning in trainings based on contraversive (n–t, orange) than with ipsiversive (t–n, green) vestibular stimulation.

To test the hypothesis that the strength of gain adaptation depends on the direction of eye movements, we used the sigmoidal stimulus during visuo‐vestibular mismatch training. Animals were trained to increase their VOR gain by rotating the visual stimulus out of phase (180°) with the vestibular stimulus and decrease their VOR gain by rotating both stimuli in phase (0°) (*n* = 35 mice) (Fig. 5 *A*; see also Supporting information, Movie S2). Interestingly, out of phase trainings with eye movements in the n–t direction (i.e. contraversive vestibular and ipsiversive visual rotation) resulted in a prominent increase of eye movements during probe trials (*n* = 11, Δ‐gain: n–t +0.37 ± 0.06; *P* = 0.04, *F* = 9.34), whereas increase trainings with movements in the t–n direction were not successful, with a trend towards gain decrease (*n* = 13, Δ‐gain: t–n −0.1 ± 0.02; *P* = 0.1, *F* = 2.4; n–t *vs*. t–n: *P* < 0.001, *t* = −11.22) (Fig. 5 *B*, top). Similar to VOR gain–increase, decrease training was more effective when the visual and vestibular stimulus rotated in phase to suppress eye movements in the n–t direction (*n* = 13, Δ‐gain: t–n −0.17 ± 0.02) than in the t–n direction (*n* = 13, Δ‐gain: n–t −0.04 ± 0.03; n–t *vs*. t–n: *P* = 0.001, *t* = 3.62). This effect was not dependent on the presence of visual feedback during the secondary segment of the stimulation (Fig. 6), because the learned responses were similar when mice were subjected to only unidirectional visual training stimulation, with the table rotating back to the starting position in the dark (gain–increase n–t: 0.37 ± 0.08, t–n: 0.02 ± 0.04; *P* = 0.079, *t* = 1.761; gain–decrease n–t: −0.02 ± 0.03, t–n: −0.17 ± 0.02; *P* = 0.18, *t* = 1.34) (Fig. 3 *B*, right). The decrease in gain was not the result of habituation (Nagao, 1983; Gutierrez‐Castellanos *et al*. 2013), because ‘training’ stimulation in the dark, without visual input, resulted in a minimal change in gain during n–t movements (Fig. 5 *B*, bottom left, grey curve). This change was different from that in the preferred, contraversive direction (*P* < 0.001, *t* = 3.76), suggesting that habituation is relatively limited for sigmoidal stimulation.

These results demonstrate for the first time that VOR gain adaptation in mice is direction‐sensitive in that the adaptation is more pronounced during both increase and decrease trainings during contraversive vestibular input. This implicates that eye movements in the preferred (i.e. n–t) direction are more prone to adapt, whereas, at the same time, the contralateral eye does not undergo obvious changes.

### Gain–increase training leads to enhancement of SSs

The findings that compensatory eye movements and related SS modulation are both optimal in the n–t direction and that learning visuo‐vestibular mismatch tasks is also optimal when the contraversive vestibular stimulus elicits eye movements in the n–t direction point towards preferred plasticity mechanisms that depend on movement direction (i.e. a principal learning direction). Lesions of the flocculus ablate the ability to adapt the VOR (Kassardjian *et al*. 2005; Shutoh *et al*. 2006), but how floccular PCs contribute to VOR adaptation and how their activity changes during learning is still under debate. Therefore, we recorded single‐unit PC activity during VOR gain adaptation (*n* = 32 cells; 17 mice) (see Supporting information, Movie S2).

**Figure 7:**
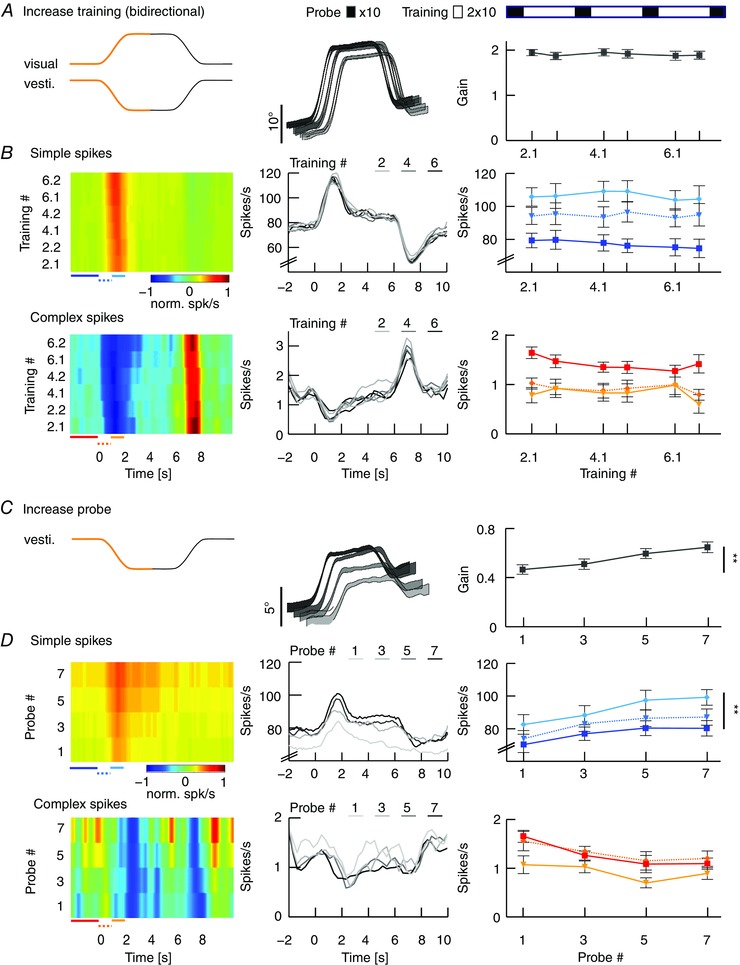
VOR gain–increase correlates with increased SS activity in probe trials *A*, VOR gain–increase training was induced by simultaneous visual and vestibular stimulations moving in opposite directions. After an initial VOR baseline measurement including 10 stimulus cycles of 12 s each (2 min), we recorded from each cell during three consecutive training blocks (total duration 20 min). Each of these training blocks consisted of two visuo‐vestibular mismatch training sessions and each session included 10 stimulus cycles (i.e. 2 × 2 min) and one VOR probe session including 10 stimulus cycles (i.e. 1 × 2 min). Only repeats with stable spiking activity were used for analysis. From the start of the gain–increase training, the eye movements of the mice compensated close to optimally for the combined amplitude of the visual and vestibular stimulation and the gains during this visuo‐vestibular mismatch stimulation did not change significantly over time (middle). Schematic drawing at the top right illustrates the order of 10 VOR probe trials in the dark (black boxes) and 20 visuo‐vestibular training trials (white boxes) for three consecutive blocks. *B*, heat maps of normalized SS and CS responses of an example PC during the training trials qualitatively (left) and average firing rates of all cells for each training block quantitatively (middle) representing unimodal and reciprocal SS and CS firing rates. Absolute firing rates (right) were determined for three intervals during the first segment of stimulation as indicated by coloured lines below heat maps of relative firing rate (left): the baseline before the stimulus (continuous lines, SSs dark blue and CSs red) compared to the accelerating and decelerating half of the stimulus (dotted and continuous line, SSs light blue and CSs light red, respectively). SS and CS activity were stable over the subsequent training sessions. *C*, as a result of the increase training sessions, eye movement amplitude in the probe trials (i.e. during vestibular stimulation in the dark only) increased from starting gains of ∼0.4 to ∼0.7 (right). *D*, similar to (*B*), but here for probe trials. Although CS activity was not affected, SS firing rate increased significantly, particularly during the decelerating phase of the initial segment and during the stationary segment of the stimulus, over the course of the training sessions (right). Data are the mean ± SEM; eye movement population data (middle) represent the mean ± SEM and spiking data are the mean values.

**Figure 8:**
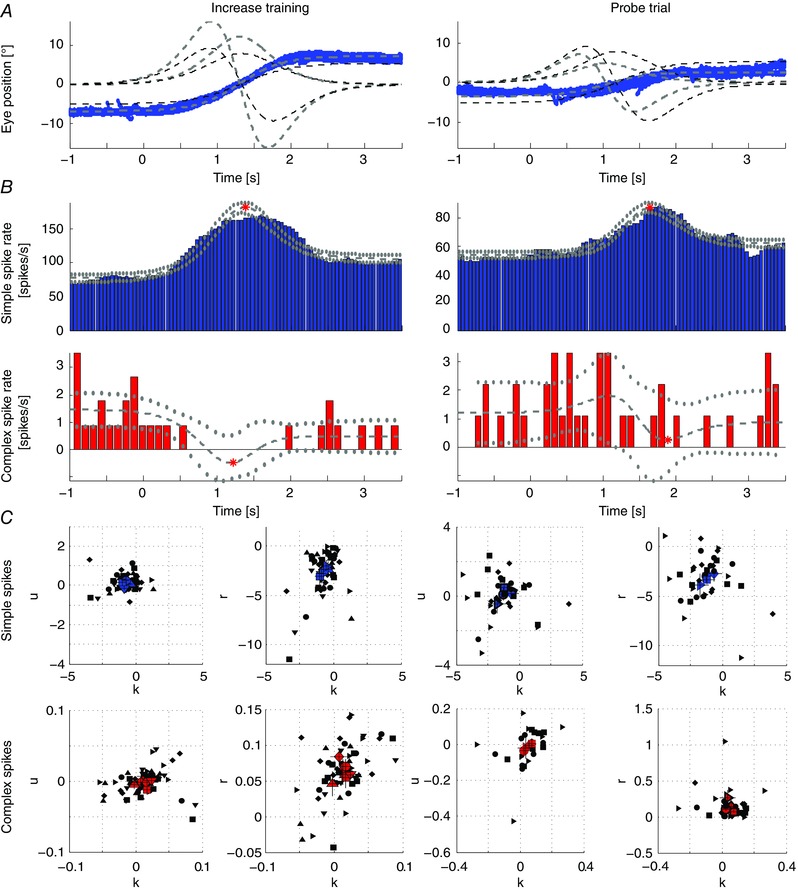
Regression of VA PC responses during increase training *A*, position, velocity and acceleration profiles of the initial segment of a sigmoidal vestibular stimulation (black) and the corresponding averaged eye movement responses of a single mouse (grey) during increase training (left) and during the first probe trial (right). Note that, during the increase training (left), the added visual stimulation results in compensatory eye movements larger than that of the vestibular stimulus alone (right). *B*, corresponding averaged SS (blue) and CS (red) activity profiles of a VA Purkinje cell during these initial segments of the training and probe trial; data were fitted using an inverse dynamics model of the eye movements (grey) (Eqn 2). *C*, results of all recorded PCs for position (*k*), velocity (*r*) and acceleration (*u*) components of SSs (top row) and CSs (bottom) during increase training (left) and probe trials (right). Data are the mean ± SEM.

**Figure 9:**
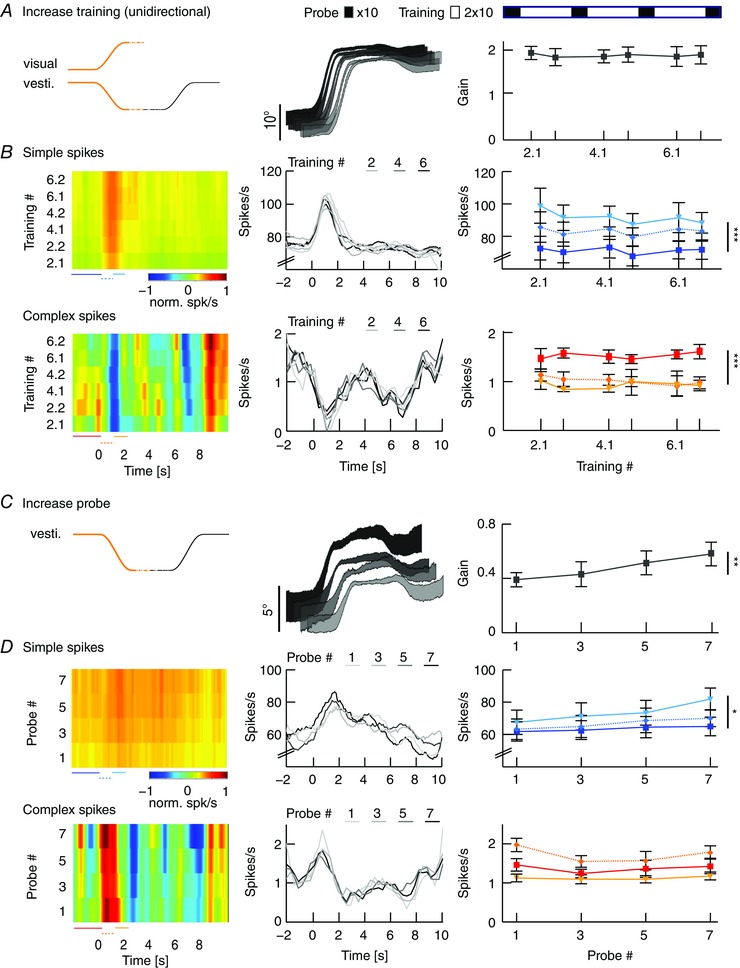
Eye movements and PC modulation during training and probe trials following unidirectional training *A*, stimulus and average eye movements during unidirectional increase training, which consisted of visual and vestibular stimuli moving in opposite directions during the initial segment of the stimulus and the vestibular stimulus rotating back without the visual stimulus present during the second segment (left). Eye movements compensated for the combined amplitude of the stimuli during the initial segment (middle) and gains did not change over time (right). *B*, heat map of normalized SS and CS responses of a representative PC (left) and average firing rates for each training block (middle). Absolute firing rates (right) were determined for three intervals during the first segment of stimulation as indicated by coloured lines below heat maps of relative firing rate (left): The baseline before the stimulus (continuous lines, SSs dark blue and CSs red) compared to the accelerating and decelerating half of the stimulus (dotted and continuous line, SSs light blue and CSs light red, respectively). Note that a significant modulation was only present during the initial segment. *C*, stimulus and average eye movements during probe trials. In probe trials, when the vestibular stimulus was delivered in the dark (left), eye movement amplitude significantly increased over the training sessions (middle, right). *D*, SS and CS responses of the same cell as in (*B*) (left) and on average for all cells (right) show that SS activity increases during vestibular stimulation over the course of gain–increase learning. Spike data on the left are the mean values. Eye movement population data in the middle and spike data on the right represent the mean ± SEM.

VOR gain was increased in the identified principal learning direction by contraversive vestibular and ipsiversive visual rotation (10° amplitude each), resulting in n–t movements of the ipsilateral eye, which compensated fully for the stimulus amplitude (eye movement amplitudes ∼19–20°) (Fig. 7 *A*, middle). In line with that previously shown in sinusoidal training paradigms (Raymond & Lisberger, 1998; Badura *et al*. 2016), the SSs showed a substantial increase in firing rate during the initial segments of the individual training cycles, during which the eyes moved into the n–t direction (Fig. 7 *B*). Concomitantly, CS firing rate decreased with a reciprocal pattern. However, displaying close to perfect eye movement compensation, neither the behavioural responses, nor the SS or CS spiking patterns changed over successive training sessions (*n* = 10, gain: *P* = 0.97, *F* = 0.99; SS: *P* = 0.9, *F* = 0.3; CS: *P* = 0.9, *F* = 0.3) (Fig. 4 *A* and *B*, right). By contrast, and as expected, over the successive VOR probe trials, eye movement gain steadily and significantly increased (*P* = 0.004, *F* = 5.69) (Fig. 7 *C*). Concomitantly, SS modulation also increased from 8 ± 1 to 20 ± 3 spikes s^−1^ (*P* = 0.004, *F* = −3.04), with a peak in modulation at the initial segments of the VOR probe cycle, which predominantly encoded velocity (Fig. 8 *A* and *B*). During the stationary segment of the stimulus, after the eye had rotated to a temporal position, SS firing maintained a plateau level suggesting encoding of position; this level increased from 71 ± 4 spikes s^−1^ in the first trial to 85 ± 5 spikes s^−1^ in the last trial (Fig. 7 *D*) (*P* = 0.045, *t* = −2.832). By contrast, CS modulation remained constant not only during training, but also during probe trials (training: *P* = 0.91, *F* = 0.299; probe: *P* = 0.98, *F* = 0.51). During the secondary segment of the training stimulus (i.e. the rotation back to the initial position) (Fig. 7 *B*, black parts of stimulus traces), SSs and CSs modulated with the reversed unimodal pattern reflecting conditions during trainings in the non‐learning direction (Fig. 5). Purkinje cell recordings during unidirectional increase training (i.e. when the light was turned off during the second segment of the training cycles; see also Fig. 6 *A*) revealed similar results (Fig. 9) (*n* = 5, unidirectional *vs*. bidirectional: gain: *P* = 0.11, *t* = 1.62; SSs: *P* = 0.38, *t* = 0.89; CSs: *P* = 0.1, *t* = 1.8), highlighting the facilitatory role of SS activity for gain–increase learning with contraversive head rotation.

### Lower VOR gains after decrease training do not correlate with changes in PC simple spike activity

**Figure 10:**
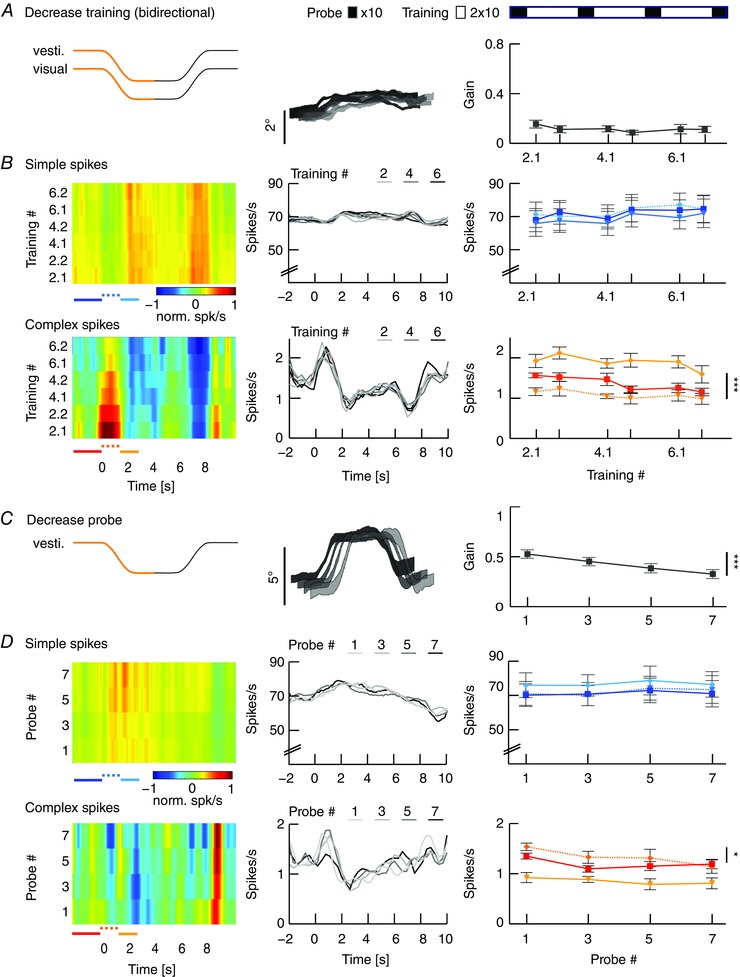
VOR gain–decrease affects CS activity during training sessions *A*, VOR gain‐decrease training was induced by simultaneous visual and vestibular stimulations that move in‐phase (left). This resulted in minimized eye movements (middle) that did not change in gain over time (right). Order of VOR probe trials (black boxes lasting 8 min) and training sessions (white boxes lasting 12 min) is illustrated above the line plots. *B*, SS and CS activity, on average, did not change during the decrease training sessions. Insets (middle) show a graphical fit of the acceleration profile of the stimulus onto the bimodal average SS and CS modulation during the initial segment of the stimulus. Absolute firing rates (right) were determined for three intervals during the first segment of stimulation as indicated by coloured lines below heat maps of relative firing rate (left): the baseline before the stimulus (continuous lines, SSs dark blue and CSs red) compared to the accelerating and decelerating half of the stimulus (dotted and continuous line, SSs light blue and CSs light red, respectively). *C*, vestibular stimulus and average eye movements during probe trials in the dark (left). Eye movement amplitude decreased over subsequent training sessions (middle), with gains starting from ∼0.4 and significantly decreasing to ∼0.26 (right). *D*, same PC as in (*B*) (left) and group averages (middle) showing that gain–decrease was reflected in CS activity but not in SS activity (right). Spike data on the left are the mean values. Eye movement population data in the middle and spike data on the right represent the mean ± SEM.

During VOR gain–decrease training in the principal learning direction, the contraversive vestibular stimulus was combined with an in‐phase visual stimulation (10° amplitude each) to suppress eye movements in the n–t direction (Fig. 10 *A*). As predicted based on the minimized level of eye movements, SS modulation was largely absent during gain–decrease training sessions, showing a slightly bimodal pattern with no significant difference in SS peak firing rates compared to rest (*n* = 11, SS rest *vs*. stimulation: *P* = 0.746, *t* = −0.325) (Fig. 10 *B*). Moreover, we observed no significant changes in eye movement gain or SS modulation across the successive training sessions (gain: *P* = 0.5, *F* = 0.9; SS: *P* = 0.59, *F* = 0.75) (Fig. 5 *B*). By contrast, during the intermittent probe trials, VOR gain values steadily and significantly decreased (*P* < 0.001, *F* = 9.82) (Fig. 10 *C*). However, we found no changes in SS firing during the probe trials over the course of the experiment (*P* = 0.92, *F* = 0.17) (Fig. 10 *D*).

**Figure 11:**
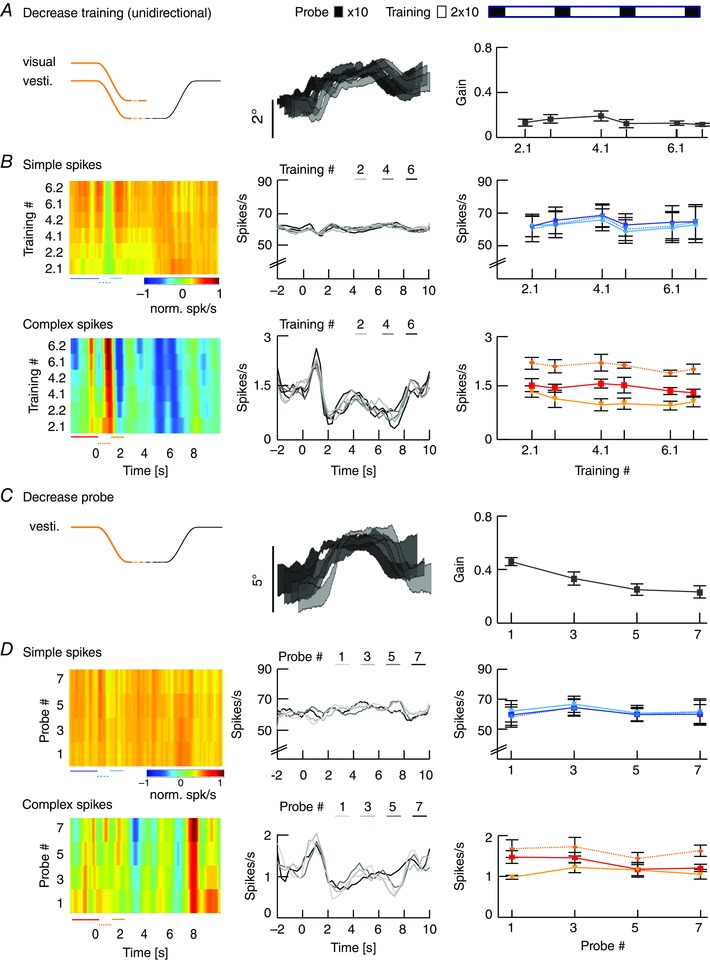
Eye movements and PC modulation during training and probe trials following unidirectional decrease training *A*, stimulus and average eye movements during unidirectional decrease training consisting of visual and vestibular stimuli moving in phase during the initial segment of the stimulus and the vestibular stimulus rotating back without the visual stimulus present during the second segment (left). Eye movements were minimized as a result of the stimuli during the initial segment (middle) and these gains did not change over time (right). *B*, heat map of normalized SS and CS responses of a representative PC (left) and average firing rates for each training block (middle). Absolute firing rates (right) were determined for three intervals during the first segment of stimulation as indicated by coloured lines below heatmaps of relative firing rate (left): The baseline before the stimulus (continuous lines, SSs dark blue and CSs red) compared to the accelerating and decelerating half of the stimulus (dotted and continuous line, SSs light blue and CSs light red, respectively). SS responses were largely absent, whereas CSs showed a similar bimodal pattern as seen in bidirectional training (Fig. 10). *C*, stimulus and average eye movements during probe trials. In probe trials, when the vestibular stimulus is delivered in the dark (left), eye movement amplitude significantly decreased over the training sessions (middle, right). *D*, SS and CS responses of the same cell as in (*B*) (left); on average, the cells (middle, right) show no changes in SS or CS activity over time. Eye movement population data in the middle and spike data on the right represent the mean ± SEM.

CS modulation during gain–decrease training sessions was also bimodal, but now with a significant peak‐to‐peak modulation (i.e. initial increase followed by a decrease; *P* < 0.001, *t* = 10.521), closely resembling the pattern of baseline VOR (complex spikes during training) (Figs 4 *C*, 8 *B* and 10 *B*). During the probe trials, CS modulation was unchanged (*P* = 0.4, *F* = 1.1), although the baseline firing frequency showed a significant decrease (*P* = 0.025, *F* = 3.76) (Fig. 10 *D*). This change in CS activity during the probe trials with bidirectional stimulation was also observed during probe trials after unidirectional training sessions with a concomitant change in gain, whereas SS activity did not change (*n* = 6, mixed model unidirectional: gain: *P* = 0.003, *F* = 9.257; SS: *P* = 0.9, *F* = 0.1; CS: *P* = 0.02, *F* = 2.91) (Fig. 11).

### Causality and reversibility support a paradigm‐dependent location of neuronal correlates

**Figure 12:**
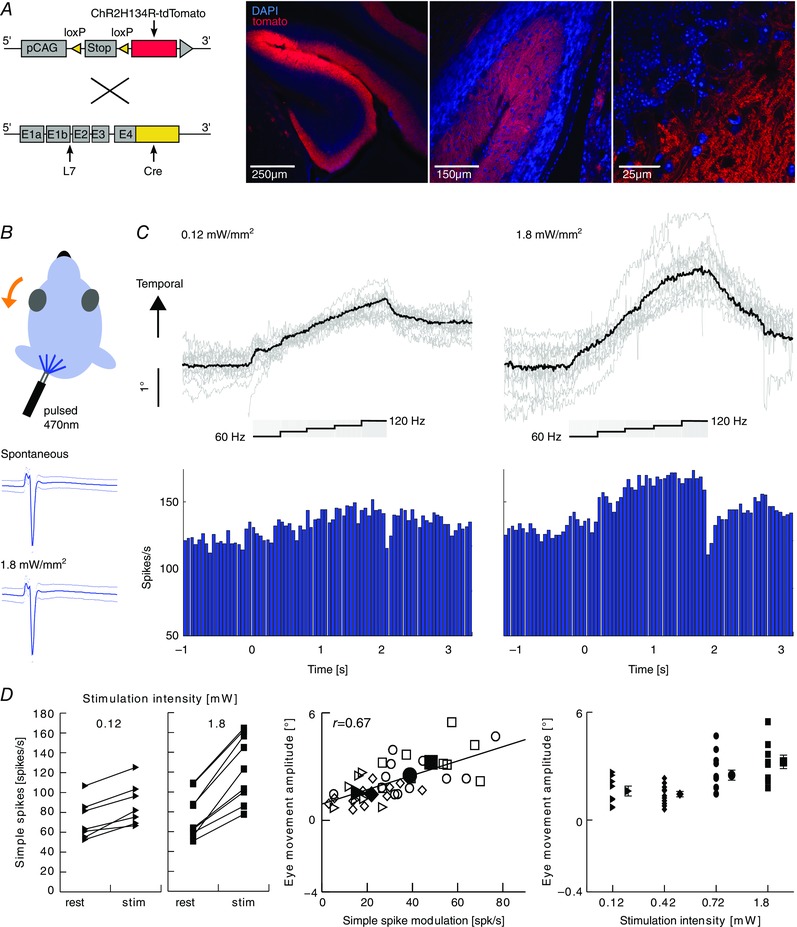
Interactions between SS activity and direction and amplitude of eye movements *A*, Purkinje cell‐specific Cre (L7‐Cre) and Cre‐recombinase‐dependent channelrhodopsin expressing (Ai27D) mice were crossed to generate mice in which the firing activity of Purkinje cells can be increased by a light stimulus (left). Confocal images of sagittal cerebellar slices of the flocculus‐parafloccular complex showed reliable expression of channelrhodopsin (red) in PCs. Blue indicates 4',6‐diamidino‐2‐phenylindole staining. *B*, selective optogenetic stimulation of PCs in the flocculus with blue light (pulsed 470 nm; five steps: 60, 80, 90, 100 and 120 Hz for 400 ms each) resulted in temporal eye movements (top). SS shapes did not differ between spontaneous and evoked activity (bottom). *C*, example eye movement traces (top, *n* = 10, black line = average) following repeated optogenetic stimulation (grey box) mimicking the naturally evoked response, raster plot (middle) and PSTH (bottom) of the SS response as a result of low (left) and high (right) light intensities (for CSs, see Fig. 13). *D*, increasing intensities resulted in increases in SS firing rates of individual cells (left) and accompanying ipsiversive eye movements (right). Optogenetically evoked changes in SS firing correlate with changes in eye movements (middle) in terms of direction and eye movement per spike, indicating a causal relationship (triangles, diamonds, circles and squares indicate 0.12, 0.42, 0.72 and 1.8 mW mm^−2^, respectively, filled symbols represent the mean values). Note that optogenetic stimulation also evoked eye movements in the vertical plane (Fig. 13).

**Figure 13:**
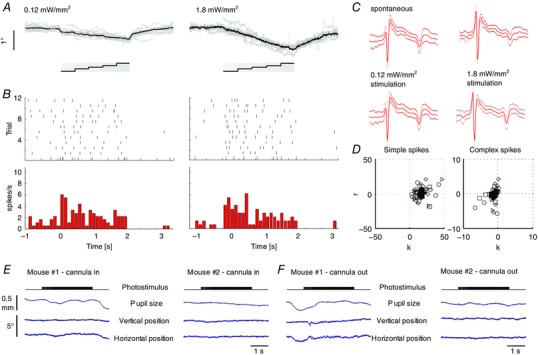
Optogenetic stimulation of floccular PCs and corresponding eye movements *A*, examples of vertical eye movements evoked by stimulation traces (bottom) at different light intensities. Stimulation traces consisted of light pulses with increasing frequencies (60, 80, 90, 100 and 120 Hz; 50% duty cycle; 450 ms duration each). Eye movements increased in amplitude with increasing light intensity and were typically diagonal, consisting of a temporal and ventral component (compare horizontal eye movements in Fig. 12 *A*). *B*, raster plots and PSTHs of CS activity corresponding to the eye movements in (*A*). *C*, average CS shapes and 95% confidence interval of the cell in (*B*) during stimulation and interstimulus intervals. Shapes of the two spike types were not different during these periods, suggesting that the optogenetically evoked CS are electrophysiologically similar to spontaneous CSs (for SSs, see Fig. 6 *B*). *D*, linear regression of the eye movement onto PC cell firing showed a strong correlation with the position component (*k*) for the four light intensities of both SS and CS firing (0.12 mW, position *k*: SSs = 19.7 ± 4.9, CSs = −1.2 ± 0.5, velocity *r*: SSs = 1.2 ± 2.2, CSs = −0.9 ± 0.3; 1.8 mW, position k: SSs = 16.5 ± 2.6, CSs = −1.6 ± 0.5, velocity *r*: SSs = −2 ± 1.5, CSs = −0.5 ± 0.2). Using a linear fit of the eye movements, the constant velocity term represents the offset of spiking, whereas the acceleration term was zero and did not contribute. *E*, control experiment in wild‐type mice not expressing the channelrhodopsin. Two mice were prepared as before and the light stimulus presented with the probe inserted in the cannula. No changes in pupil size or position (vertical, horizontal) were observed. *F*, as in (*E*), where light stimulus was presented with probe retracted from the cannula and hovering close to the entry site.

To determine whether the changes in SS modulation are sufficient to explain the differences in eye movement amplitude during VOR adaptation, we attempted to replicate the responses to a sigmoidal stimulus, using selective optogenetic manipulation of PCs in L7/cre‐Ai27 mice in the dark without vestibular stimulation (*n* = 20 cells; four mice) (Fig. 12 *A*). Unilateral stimulation of floccular PCs with blue light pulses resulted in SSs and CSs with a normal shape (Figs 12 *B* and 13 *A–D*). Increasing stimulus frequency induced concomitantly an increase in SS firing rate and n–t eye movements (Fig. 12 *C*), mimicking those evoked by natural vestibular or visual stimulation. The SS firing rate and pupil position changed proportionally to the light intensity (0.12, 0.42, 0.72, 1.8 mW mm^−2^) (Fig. 12 *D*). To ensure that light stimulation was a result of activation of PCs and not stimulating the eye directly, we performed the same experiment in wild‐type mice not expressing the channelrhodopsin. Neither pupil size, nor eye position changed during the light stimulus in these mice (Fig. 13 *E* and *F*). The change in spike rate per degree of eye movement was significantly higher during optogenetic stimulation than that during VOR adaptation (7 ± 2 *vs*. 4 ± 3 spikes/s/degree, respectively) (two‐tailed *t* test: *P* = 0.02, *t* = 2.52), possibly reflecting the fact that the basic drive from the primary vestibular afferents to the second‐order vestibular neurons is present during VOR adaptation, but not during optogenetic stimulation (Fig. 1 *A*) and/or that natural vestibular stimulation exerts bilateral effects, whereas our optogenetic stimulation was provided unilaterally. In conjunction with the increase in SS activity following natural training stimulation, these data suggest that SS activity can code for gain–increase adaptation contributing to movement‐direction selective learning.

**Figure 14:**
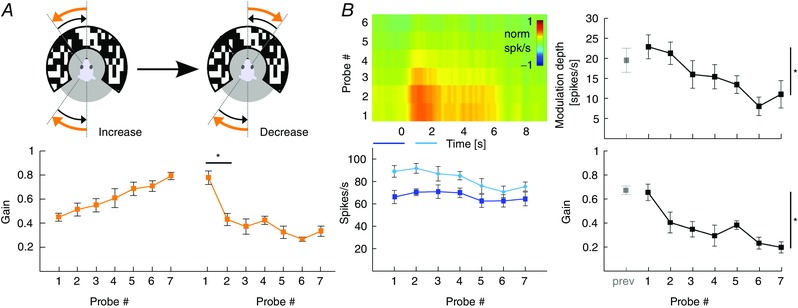
Neuronal correlates for VOR adaptation involve the cerebellar cortex and targets downstream of PCs *A*, VOR gain–decrease training can reverse adapted changes in eye movement gain as a result of increase training. Icons (top) depict the paired paradigm. Note the sharp drop in gain after the first decrease training block (bottom). Data from behavioural experiments. *B*, heat map of simple spike activity of an example cell (left top) showing that the increase in SS firing after gain–increase training returns to near baseline levels during decrease training (compare with Figs 4 *D* and 5 *D*). Averaged firing rates of all recorded cells (*n* = 8) prior to (dark blue) and during (light blue) stimulation (left bottom). Eye movement gains, as in (*A*), decreased with an initial fast drop followed by a slower decrease (right bottom), whereas the depth of simple spike modulation (right top) returned more gradually to baseline levels (grey data point indicating depth of modulation after increase training, cf. Fig. 7 *D*). Data are the mean ± SEM.

Gain–increases and gain–decreases are reversible when induced with sinusoidal stimulation (Boyden *et al*. 2004; Broussard *et al*. 2011). Interestingly, the reversibility is asymmetric, implicating that the two paradigms depend on different processes (Boyden *et al*. 2004), which could be in different locations. Given that, with sigmoidal stimulation, SS modulation was increased over consecutive probe trials following VOR gain–increase training, and that there was no significant change in SS activity upon VOR gain–decrease training in the probe trials, it could be hypothesized that the learning abilities might ultimately be blocked as a result of saturation. Alternatively, the gain–decrease training could actively extinguish the changes acquired during gain–increase training, in effect at least in part ‘cleaning the sheet’ for new trainings. To test this, we performed an additional set of behavioural experiments, in which mice (*n* = 6) were first subjected to VOR gain–increase training, immediately followed by a decrease training, both in the principal learning direction. The first block of decrease training ablated the effect of seven blocks of increase training (total of 98 min), significantly reducing VOR gains to baseline levels (first *vs*. second probe trial: *P* = 0.039, *t* = 2.47) and below (baseline *vs*. last decrease probe trial: *P* = 0.034, *t* = −2.564) (Fig. 14 *A*). During the remaining decrease training, eye movement gains further decreased, comparable to the effect of VOR gain–decrease training alone (Fig. 5 *B*). To assess SS dynamics during this extinction, we next recorded PC activity related to the decrease training (*n* = 8) after the mice had received an increase training (Fig. 14 *B*). Although eye movement gains, similar to before, decreased rapidly after the first decrease training, SS modulation depth declined more gradually from a potentiated peak firing rate back to baseline levels (first *vs*. second probe: *P* = 0.64, *t* = −0.48; all probes: *P* = 0.03, *F* = 3.88) (Fig. 14 *B*) than could be expected based upon the rapid changes in eye movement gain at the initial stage. This partial discrepancy between behaviour and neuronal signals may reflect the differential loci for unlearning VOR gain–increase and learning VOR gain–decrease, which is also suggested by the absence of a change in SS rate following VOR gain–decrease training alone.

## Discussion

**Figure 15:**
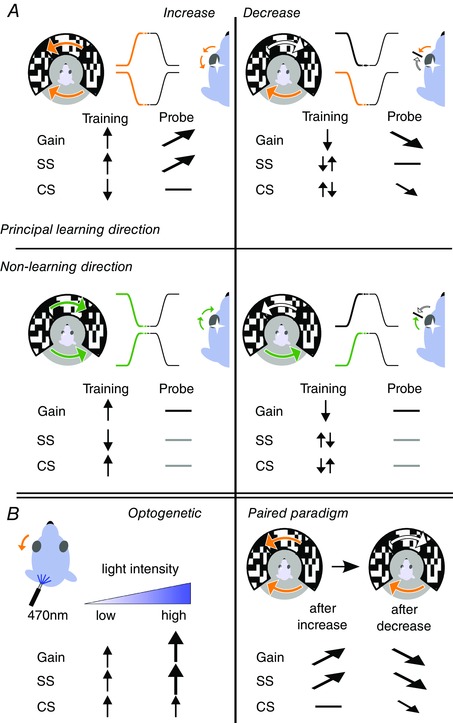
VOR adaptation and its neural correlates are paradigm and direction specific *A*, VOR adaptation and related spiking changes during gain–increase (left) and gain–decrease (right) adaptation paradigms in the principal (top) and non‐learning (bottom) direction. The gain of the VOR evoked by contraversive rotation could be adapted, increased or decreased, whereas that evoked by ipsiversive rotation could not. During gain–increase training in the principal learning direction, simple spike and complex spike rates increased and decreased, respectively, and the simple spike firing rate during probe trials after training increased significantly, in line with the VOR gain. Conversely, complex spike and simple spike modulation during gain–decrease training was more complex, revealing a bimodal CS pattern during training and a CS reduction during the probe trials, whereas simple spike rates did not change over time in probe trials. *B*, ipsilateral optogenetic stimulation and simultaneous recordings of PCs in the flocculus evoked an increase in SS and CS firing and n–t eye movements. Increasing light intensities led to increasing PC activity and eye movement amplitudes, effectively simulating the behavioural and neuronal changes observed as a result of visuo‐vestibular mismatch, gain–increase training (left). A paired paradigm showed that simple spike activity potentiated by gain–increase training, unlike that in naïve mice, can be actively suppressed by gain–decrease training. This discrepancy and the rapid drop in eye movement gain compared to the gradual decrease in simple spike activity point towards the potential involvement of downstream areas in the decrease of eye movement gain (e.g. the vestibular nuclei).

Directionality is an inherent feature of the compensatory eye movement system and manifests itself in the lateralization of the cerebellum and the mirrored preferred axes of modulation for climbing fibre activity of PCs in the various zones of both flocculi (Wylie *et al*. 1994; Schonewille *et al*. 2006). To test the hypothesis that cerebellar learning depends on movement direction, we investigated to what extent compensatory eye movements and VOR gain–increase and gain–decrease adaptation are direction‐specific. Behavioural results showed that visual input generated a direction‐selective difference in eye movement gains and delays, with larger gains and longer delays for eye movements in the n–t direction. Learning efficacy was optimal when the vestibular stimulus moved contraversively, which coincides with the preferred n–t movement direction for visually driven compensatory eye movements. In addition, our electrophysiological data showed that SS activity during probe trials could only be correlated with eye movements during VOR gain–increase adaptation in the n–t direction. Moreover, albeit in a reciprocal fashion, CS activity during probe trials was also best correlated with eye movements during VOR gain–decrease adaptation in the same preferred direction (Fig. 15 *A*). Indeed, taken together, these results point towards a principal learning direction for eye movement adaptation.

To the best of our knowledge, this is the first study correlating SS and CS activity with eye movement adaptation using sigmoidal trainings and probe trials during complete cycles of learning. This experimental set‐up allowed quantitative identification of learning rules and direction selectivity for both gain–increase and gain–decrease (Nguyen‐Vu *et al*. 2013; Kimpo *et al*. 2014). Interestingly, gain–increase training was exclusively successful when the visual stimulus moved the eye into the preferred n–t direction, minimizing the activity of the climbing fibres. These data are in line with our working hypothesis that potentiation rather than depression mechanisms drive VOR learning (Schonewille *et al*. 2010, 2011; Gutierrez‐Castellanos *et al*. 2017), because LTP at the parallel fibre to PC synapse and enhancement of PC intrinsic plasticity are facilitated by a reduction in climbing fibre activity (Gao *et al*. 2012). Importantly, our optogenetic experiments demonstrated that the increase in SS activity is not only necessary, but also sufficient to increase the gain (Figs 12 and 15 *B*). Similarly, the shorter delays for eye movements in the temporo‐nasal (t–n) direction may be related to the rapidly and synchronously activated CS during contraversive movements, because a lack of olivary gap junctions induces a short delay during such visual compensatory eye movements (Kistler *et al*. 2002). Other forms of plasticity in the cerebellar cortex may also influence SS activity of PCs and thereby gain–increase learning (Gao *et al*. 2012). For example, these include those engaging the molecular layer interneurons, which directly control the rate and regularity of SS activity (Miyashita & Nagao, 1984; Gao *et al*. 2012). The inhibitory interneurons, as well as the plasticity mechanisms that control their activity, are probably also relevant for the inversion of the vestibular signal that occurs between the vestibular organs and the Purkinje cells (Miyashita & Nagao, 1984). Contraversive head rotation decreases activity in the vestibular ganglion neurons but correlates with an increased firing rate in Purkinje cells (Fig. 1 *A*). Hence, suppression of the inhibitory input, which is indeed facilitated during the absence of climbing fibre activity (Gao *et al*. 2012), may contribute to VOR gain–increase learning, whereas the absence of effects on SS firing rate during gain–decrease training suggests that changes in inhibition in this form of adaptation are absent or nullified by concomitant changes in excitatory input or intrinsic properties. In line with this, mutant mice lacking GABAγ2 receptor subunits at their molecular layer interneuron to PC synapse or mutants lacking the potassium chloride transporter KCC2 specifically in their PCs, both of which suffer from impaired inhibition onto their PC, show virtually normal gain–decrease training, whereas gain–increase learning and phase reversal learning are strongly affected (Wulff *et al*. 2009; Gao *et al*. 2012; Seja *et al*. 2012). Thus, the changes in SS firing that we observed during gain–increase training may be potentially enhanced by net changes of inputs from both excitatory and inhibitory inputs (Badura *et al*. 2013; Barmack & Yakhnitsa, 2013), although their functional impact during gain–decrease learning appears to be rather limited.

A dichotomy between gain–increase and gain–decrease has been suggested previously, although the evidence for the underlying locations and processes is inconclusive (Boyden *et al*. 2004; Boyden *et al*. 2006; Schonewille *et al*. 2010; Titley and Hansel, 2016; Gutierez‐Castellanos *et al*. 2017). Presumably, plasticity in the vestibular nuclei downstream contributes substantially to gain–decrease learning (Gittis & du Lac, 2006; McElvain *et al*. 2010; Jamali *et al*. 2016). Indeed, the gain–decrease paradigm did not result in persistent changes in SS activity, but only in changes in CS activity that may influence plasticity in the vestibular and cerebellar nuclei (Pugh & Raman, 2006; De Zeeuw *et al*. 2011) (Fig. 15 *A*). These data are in line with the observation that mutants, in which the majority of granule cell output is impaired and both LTD and LTP are impaired, can still successfully complete the gain–decrease but not the gain–increase paradigm (Galliano *et al*. 2013). In addition, plasticity at the level of vestibular nuclei during gain–decrease learning may also explain why gain–increase effects are rather specific for the training frequency (Lisberger *et al*. 1983), whereas gain–decrease appears to be more generalized over a wider range of vestibular input frequencies (Kimpo *et al*. 2005). Finally, the data of the present study are also in line with the behavioural and spike activity phenotypes of various mutants during phase reversal of the VOR, which requires initially an extension of the gain–decrease paradigm followed by an increase in gain, ultimately moving the eye opposite to the natural reflex (Clopath *et al*. 2014; Badura *et al*. 2016).

The notion that changes in SS activity of floccular PCs could be directly responsible for the adaptive response during VOR adaptation has been proposed previously (Blazquez *et al*. 2003; Nguyen‐Vu *et al*. 2013), as has a contribution of the vestibular nuclei (Lisberger, 1994), although, in the present study, we show for the first time that this activity, as well as the level of learning, is related to the direction of the eye movement. Optogenetically driven floccular SS activity during contraversive (but not ipsiversive) vestibular input indeed resulted in a higher VOR gain (Nguyen‐Vu *et al*. 2013). By contrast, climbing fibre activation during contraversive head movement had no effect (Nguyen‐Vu *et al*. 2013), arguing against a role for climbing fibre‐dependent plasticity during VOR gain‐decrease, which is in line with our observations. Kimpo *et al*. (2014), on the other hand, observed an effect on PC activity during a gain–increase training paradigm that was assumed to occur during ipsiversive head movements when climbing fibres are activated. However, eye movement behaviour was not recorded during these PC recordings and the current data raise doubt as to whether the gain–increase adaptation occurred fully. It should be noted that, unlike the present study, the majority of previous studies used continuous sinusoidal vestibular input, hampering the analysis of direction‐specific components, and/or the previous studies did not combine behavioural analysis with simultaneous PC recordings.

Our experiments in which we investigated gain–decrease training following gain–increase training (i.e. the decrease in gain occurred following an initial enhancement of SS firing rates) (Fig. 15 *B*), revealed that gain–decrease adaptation and changes in SS modulation follow different dynamics, even when both eye movement gain and SS activity are bound to decrease. Although the gain had already dropped to baseline levels after one training session, SS modulation gradually declined over several training sessions but never dropped below baseline, once more indicating that the locus for VOR gain–decrease learning resides partly somewhere downstream of PC output. Nevertheless, proper PC activity may also be required for successful VOR gain–decrease training because it is impaired after flocculectomy (Nagao, 1983).

It is interesting to note that other laterally eyed animals such as zebrafish show a similar pronounced asymmetry in directional sensitivity of their optic system (Feierstein *et al*. 2015). Moreover, even though direction‐selectivity remains to be investigated at a more detailed level, several findings point towards similar mechanisms in frontal eyed animals. For example, in non‐human and human primates, unidirectional rotations around the vertical axis also resulted in asymmetric gain changes towards the adapted but not un‐adapted side (Ushio *et al*. 2011; Migliaccio & Schubert, 2013). Rotations around the horizontal axis in humans appear to have a direction‐specific preference for downward movements (Akao *et al*. 2007; Bonnet *et al*. 2013; Ke *et al*. 2013) that may coincide with increased SS activity in floccular horizontal‐axis zones (Hirata & Highstein, 2000; Hirrata & Highstein, 2002). It would be interesting to determine to what extent floccular PC activity is also concomitantly and selectively enhanced in frontal eyed animals during ipsiversive eye movements. If this correlation holds throughout all vertebrate species, it might be hypothesized that such control systems provide evolutionary advantageous control over explorations of the ipsilateral visual field and concomitantly ipsilateral motor control, such as limb movements. After all, with such a configuration, the cerebral cortical control systems involved, such as the frontal eye fields, primary and secondary visual cortices, as well as the cortical areas involved in limb control, all develop on the same side of the brain, offering optimal opportunities for integration from a neuro‐anatomical point of view.

The asymmetry in learning mechanisms for different directions might depend partly on the different types of cerebellar zones involved. PCs in floccular zones are predominantly, if not exclusively, zebrin‐positive and thus have a relatively low SS firing rate (Fujita *et al*. 2014; Zhou *et al*. 2014). Thereby, they are probably more prone to be potentiated by their parallel fibre input (Wang *et al*. 2011). By contrast, PCs in the zebrin‐negative zones, such as those controlling eyeblink conditioning (Mostofi *et al*. 2010), have relatively high SS firing rates and are probably more prone to be suppressed (Wadiche & Jahr, 2005; ten Brinke *et al*. 2015). Interestingly, in cerebellum‐dependent eyeblink conditioning, the learning related changes that occur in PCs are indeed in the opposite direction (i.e. SS firing is suppressed in a time‐locked fashion to the conditioned eyeblink response) (Jirenhed *et al*. 2007; Wetmore *et al*. 2014; ten Brinke *et al*. 2015). Taken together, these studies and the results of the present study suggest that potentiation and suppression of SS activity could be module‐dependent, as a general feature intrinsic of cerebellar functioning (De Zeeuw and Ten Brinke, 2015).

## Additional information

### Competing interests

The authors declare that they have no competing financial interests.

### Author contributions

KV, LP, MS and CIDZ designed, performed and analysed the behavioural experiments. KV, MS, BW and CIDZ designed, performed and analysed the electrophysiological and optogenetic experiments. KV, MS and CIDZ wrote the manuscript. CIDZ supervised the project. All authors approve of the final version of the manuscript, and agree to be accountable for all aspects of the work in ensuring that questions related to the accuracy or integrity of any part of the work are appropriately investigated and resolved, and all persons designated as authors qualify for authorship, and all those who qualify for authorship are listed.

### Funding

This work was supported by the Dutch Organization for Medical Sciences (CIDZ), Life Sciences (CIDZ and MS) and Social and Behavioural Sciences (CIDZ), Erasmus University Fellowship (MS), ERC‐Starter (MS), ERC‐adv and ERC‐POC, CEREBNET, and C7 programs of the EU (CIDZ).

## Supporting information

Disclaimer: Supporting information has been peer‐reviewed but not copyedited.

**Movie S1. Recording conditions during baseline compensatory eye movements**
Visual and vestibular stimulation as well as eye movements and PC recordings are shown for OKR, VVOR and VOR. Note that, for the purpose of the movie, the light conditions were changed. Under experimental conditions, there were no other light sources than the projection system.

**Movie S2. Recording conditions during VOR decrease and increase training**
Combined visual and vestibular stimulation for gain‐decrease and gain‐increase training. Probe trials were executed as can be seen from Movie S1 during VOR. Note that, for the purpose of the movie, the light conditions were changed. Under experimental conditions, there were no other light sources than the projection system.
